# Sequestration of P fractions in the soils of an incipient ferralisation chronosequence on a humid tropical volcanic island

**DOI:** 10.1186/s40529-021-00326-5

**Published:** 2021-12-02

**Authors:** Chih-Yu Chiu, Ian Baillie, Shih-Hao Jien, Liam Hallett, Stephen Hallett

**Affiliations:** 1 Biodiversity Research Center, Academia Sinica, 11529 Taipei, Taiwan; 2 School of Water, Energy and Environment, Cranfield University, MK43 0AL Cranfield, UK; 3 Department of Soil and Water Conservation, National Pingtung University of Science and Technology, 912-01 Pingtung, Taiwan; 4 Department of Life Sciences, Centre for Synthetic Biology and Innovation, Imperial College London, SW7 2AZ London, UK

**Keywords:** Soil P, Hedley fractions, Sesquioxides, Chronosequence, Taiwan

## Abstract

**Background:**

Phosphorus (P) is the limiting nutrient in many mature tropical forests. The ecological significance of declining P stocks as soils age is exacerbated by much of the remaining P being progressively sequestered. However, the details of how and where P is sequestered during the ageing in tropical forest soils remains unclear.

**Results:**

We examined the relationships between various forms of the Fe and Al sesquioxides and the Hedley fractions of P in soils of an incipient ferralitic chronosequence on an altitudinal series of gently sloping benches on Green Island, off the southeastern coast of Taiwan. These soils contain limited amounts of easily exchangeable P. Of the sesquioxide variables, only Fe and Al crystallinities increased significantly with bench altitude/soil age, indicating that the ferralisation trend is weak. The bulk of the soil P was in the NaOH and residual extractable fractions, and of low lability. The P fractions that correlated best with the sesquioxides were the organic components of the NaHCO_3_ and NaOH extracts.

**Conclusions:**

The amorphous sesquioxides, Fe_o_ and Al_o,_ were the forms that correlated best with the P fractions. A substantial proportion of the labile P appears to be organic and to be associated with Al_o_ in organic-aluminium complexes. The progression of P sequestration appears to be slightly slower than the chemical and mineralogical indicators of ferralisation.

## Background

Phosphorus (P) is the single most limiting nutrient in many mature tropical forests (Vitousek 2004). Even where N and cationic nutrients are important, they are often co-determinants with P (Baillie et al. 1987; Wright et al. 2011). P is relatively stable in most soils, and can even accumulate in the organic layers of some forest soils, such as in humid subtropical subalpine Taiwan (Shiau et al. 2018; Wu and Chen 2005). However, prolonged and intense weathering, leaching and erosion generally deplete P stocks, although slowly in most cases (Vitousek 2004). The ecological significance of declining P stocks as soils age is exacerbated by much of the remaining P being progressively sequestered in stable complexes and becoming less labile. Shortage of P can significantly hinder the cycling of other nutrients, as in montane soils in Borneo, where it inhibits the decomposition of organic matter and induces N deficiency (Kitayama et al. 2004).

Many studies have shown that iron and aluminium are involved in the immobilisation of P in intensively weathered tropical soils, either through co-precipitation as Fe and Al phosphates or by sorption onto the variably charged surfaces of particles of various free oxides and hydroxides (the ‘sesquioxides’) (Metzger 1941; Ghani and Islam 1946; Dubus and Becquer 2001). The sesquioxides are residual weathering products, and accumulate as aluminosilicate minerals are progressively weathered (Blume and Schwertmann 1969; Nagatsuka 1972). The increasing quantities and crystallinities of sesquioxides mean that soil capacity to sequester P often increases with age (Walker and Syers 1976). As well as the sesquioxides, stable fractions of the soil organic matter can also immobilise P by sorption and incorporation (Yusrani 2010).

In their summary of the general decrease in P lability with soil age, Walker and Syers (1976) depicted most inorganic P being finally occluded and non-labile within accreting crystalline sesquioxide particles. The organically sequestered P is shown as increasing to a broad mid-age maximum, but then declining with advancing pedo-senility. This paradigm has clarified general trends in P dynamics during the ageing of soils in tropical forests and elsewhere, and has been a useful start point for much subsequent detailed research (Vitousek 2004; Richter et al. 2006; Daniela et al. 2010; Turner and Condron 2013).

Other studies have focused on the contributions of the different sesquioxides as sites for P sorption and immobilisation, particularly the relative effects of the amorphous (oxalate extractable, Fe_o_ and Al_o_) and free forms (citrate-dithionite-bicarbonate extractable, Fe_d_ and Al_d_) (Cajuste et al. 1994; Syers et al. 1971). Some have examined how the quantities and rates of sorption from solutions of known initial P contents are related to various sesquioxides. The findings are variable, as the effects can depend on the lithology of soil parent materials. Adejumo and Omueti (2016) found that P sorption was best correlated with Fe_o_ in soils from sedimentary rocks in Nigeria, whereas the both amorphous and crystalline forms were involved in soils derived from Basement Complex granites and gneisses. However, it often appears that amorphous Fe_o_ and Al_o_ are more active than the crystalline forms (Singh and Gilkes 1991; Udo and Uzu 1972) found both amorphous (Fe_o_ and Al_o_) and crystalline (Fe_d_–Fe_o_ and Al_d_-Al_o_) forms were active sorbents in Nigeria, but Al_o_ was the most active. In contrast, Karim and Adams (1984) found that sorption was better correlated with Fe_d_ and Al_d_ in a toposequence of soils in Malawi. They also noted that kaolinite accounted for up to one quarter of the total P sorption, especially in midslope soils. Saunders (1965) concluded that P sorption in topsoils was most closely related to organic matter in topsoils, but that various Fe and Al sesquioxides were more significant in subsoils, and that sorption initially increased with weathering intensity, but declined in highly weathered soils. Bortoluzzi et al. (2015) found that goethite and ferrihydrite were more active in P sorption than more crystalline and less hydrated hematite and gibbsite in an altitudinal sequence of basaltic Ferralsols in Southern Brazil.

An alternative approach is to examine the loci and lability of the P once it is sorbed, using Hedley-type P fractionation. As with the sorption studies, fractionation results can vary with the lithology of the soil parent materials (Daniela et al. 2010). Maranaguit et al. (2017) found that most of the P in highly weathered soils under different land uses in Indonesia was inorganic, and that some P in the putatively less labile Hedley P fractions was available for uptake by rubber and oil palm crops. Agbenin (1994) fractionated P in two toposequences in Brazil: one on homogenously high-P parent material and one on heterogenous parent materials. He concluded that P was immobilized in the mid- and lower slope soils of both toposequences by weathering and occlusion in the accreting amorphous sesquioxides. Guo et al. (2000) found that NaHCO_3_-P and some residual P were the main sources depleted by exhaustive cropping on less weathered soils, but NaOH-P was accessed more in highly weathered soils. Baumann et al. (2020) found that about a quarter of the total P in the topsoils of three imperfectly drained soils in Northern Germany was labile (i.e. water + resin + bicarbonate extractable) and much of the rest was sorbed on sesquioxides, including a substantial proportion of occluded P, especially in the subsoils. Dubus and Becquer (2001) found that P was intensely adsorbed by the wholly sesquioxidic clay fractions in Ferralsols in New Caledonia, and that P availability was positively related to organic matter.

In this study we use Hedley-type fractionation to examine the effects of the incipient ferralisation and the sesquioxides on the forms, quantities and retention of soil P in relation to sesquioxides and organic matter in the soils of a topo- and chrono-sequence in the tropical environment of Green Island, Taiwan. An earlier pedogenic study showed that the highest and oldest members of this sequence are weakly ferralitic, with more intense weathering and leaching and fewer argillic features than the younger soils downslope (Jien et al. 2016). We hypothesise that the older soils have a greater capacity for P sorption, that the sorbed P becomes less labile as it becomes more occluded, and that these changes are associated with increasing contents and crystallinities of sesquioxides as the soils age.

## Methods

### Study site and soils

**Fig. 1:**
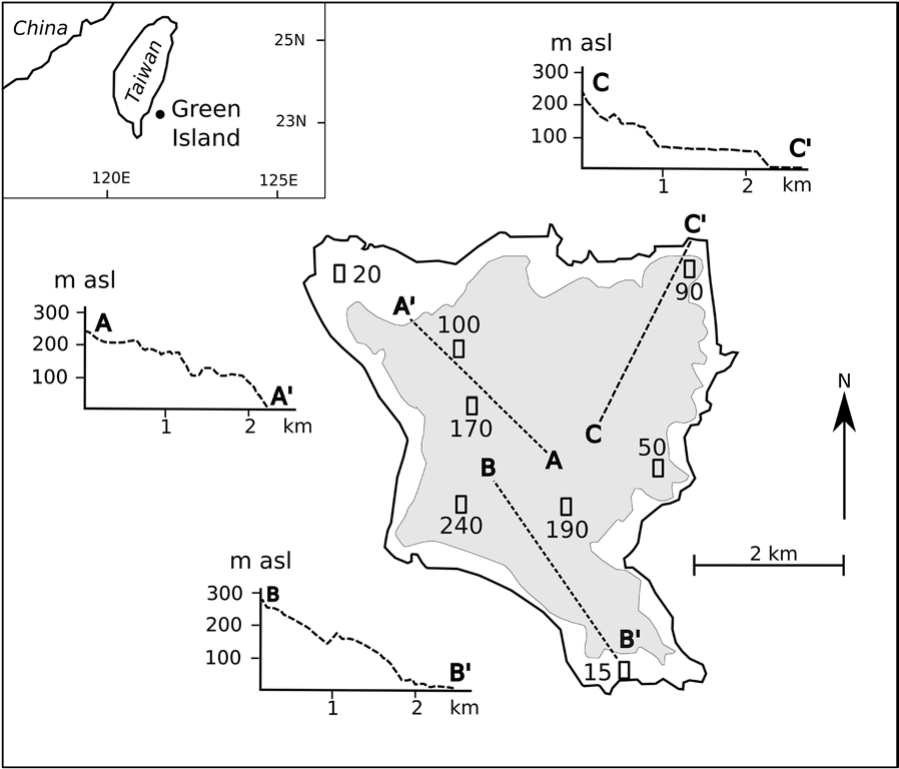
Location of study sites on Green Island, Taiwan

Green Island, also known as Ludao, (22.6 N, 121.4 E), lies about 30 km off the south-eastern coast of Taiwan (Fig. 1); it has a mean temperature of 23.5 °C and mean precipitation of about 2500 mm. The original forest vegetation in this island has been heavily disturbed by wildfire and human activities. A large-scale afforestation effort was conducted in the 1960s, and consequently most of the area is now covered with secondary broadleaved forest. The dominant tree species in these broadleaf forests are *Ardisia sieboldii*, *Schefflera octophylla*, and *Ficus nervosa.*The island is underlain by Miocene and Pliocene andesitic-basaltic pyroclastic deposits and lava flows. The topography consists of irregular low hills with an altitudinal series of discontinuous, gently sloping benches (Fig. 1). There has been no volcanic activity for about two million years, but seismic activity continues and the benches appear to be formed in still- stands of spasmodic seismic uplift (Huang et al. 2012). Reefs developed along the coast during Quaternary still-stands, and now form coral benches at about 10 and 30 m above sea level (Fig. 1). These are distinct from the higher benches located on volcanic parent materials. Chen and Liu (1992) estimated the ages of the seven terraces they identified on Green Island as: about 80 ka for the 245–255 m level, 70 ka (190–200 m), 60 ka (165–175 m), 50 ka (140–150 m), 40 ka (80–90 m), 33∼35 ka (20–40 m) and *< *5.5 ka (2–15 m). Progression in the degree of weathering of the benches with altitude has given rise to a chrono-sequence of soils of increasing ferralisation (Jien et al. 2016).

P sorption in the coral-derived soils on the lower benches is not considered in detail, as it is determined by calcareous reactions, rather than by sesquioxide sorption. In the sesquioxidic soils on the benches at 50 m and above, pH and base saturation decline with altitude and age. The subsoil matrix became more intensely red, with Munsell 2.5 YR, rather than 5 YR, hues predominant. Transient textural B horizons developed at intermediate altitudes but fade in the older soils upslope. The detail of pedological observation at each site was described elsewhere (Jien et al. 2016). They interpreted the changes as a trend of initial waxing and then waning of argillisation, through to incipient ferralisation. Taxonomically, the non-coral soils progress from Eutric Cambisol, through Acrisol, to incipient Ferralsol (FAO 2014), i.e. Eutrudept - Udalf - Udult - Udox in Soil Taxonomy (Soil Survey Staff 2014).

The soil on the 100 m bench appears to be anomalously eutrophic, with elevated pH and base saturation. This was attributed either to aeolian inputs of coral sand from the coast and lowest benches, or to a more mafic inclusion within the volcanic deposits.

### Sesquioxide fractionation

The sesquioxide fractionations are for a single sample from each horizon. We determined total Fe and Al (Fe_t_ and Al_t_) with an X-ray fluorescence (XRF) analyzer (Rigaku ZSX Mini II XRF Analyzer, Japan). Free sesquioxides (Fe_d_ and Al_d_) were extracted with dithionite-citrate buffered with bicarbonate (Mehra and Jackson 1960). Amorphous sesquioxides (Fe_o_ and Al_o_) were extracted with ammonium oxalate at pH 3.0 (McKeague and Day 1966). The Fe and Al contents of the extracts were assayed by inductively coupled plasma atomic emission spectroscopy (ICP–AES) (JY124, Horiba Jobin–Yvon, France). Fe_d_-Fe_o_ and Al_d_-Al_o_ were taken as the crystalline fractions of the sesquioxides (Nagatsuka 1972; Maejima et al. 2002). The activity (Blume and Schwertmann 1969) or crystallinity (Maejima et al. 2002) ratios of the free sesquioxides were estimated by (Fe_d_-Fe_o_)/Fe_d_ and (Al_d_-Al_o_)/Al_d_.

### P fractionation

P was fractionated for duplicate samples from each horizon, using a Hedley sequence of progressively more aggressive extractants (Table 1) applied sequentially to the same sample (Hedley et al. 1982; Levy and Schelsinger 1999). Before the sequential extractions, we determined the total P (P_t_) by digesting a separate sample in concentrated H_2_SO_4_, 30% H_2_O_2_ and MgCl_2_ at 300 °C. The first stage in the sequential extraction removed labile P with a dilute salt (0.1 M KCl), rather than a resin (Elliot et al. 2002; Olila et al. 1997). The moderately labile P was extracted from the residue with 0.5 M NaHCO_3_. The residue from that was extracted with 0.1 M NaOH, for the P that is perceived to be sorbed on sesquioxides and stable organic matter and of low lability. We used the procedure of Tiessen and Moir (1993) to differentiate the inorganic and organic forms of P in both of the NaHCO_3_ and NaOH extracts. We first assayed initial P and took them as the inorganic (P_i_) components. We digested the extracts at 200 °C with concentrated H_2_SO_4_ and 30% H_2_O_2_ to solubilise the organic P. The assayed P in these digests was taken as the total P for that fraction, and the organic P (P_o_) was estimated by subtraction of P_i_. The P in apatite or associated with calcareous particles was extracted with 1 M HCl. The final extraction was for residual P (P_r_) using concentrated H_2_SO_4_ and 30% H_2_O_2_ and was aimed at the occluded P with severely restricted or zero lability. The P concentrations in all extracts and digests were assayed colourimetrically by the malachite green procedure (Lajtha et al. 1999). The sums of the extracts exceed the initial P_t_ values throughout, giving apparent extraction values in excess of 100%. This suggests that the initial non-sequential XRF determination of total P (P_t_) from the untreated fine earth was only partially effective.

**Table 1:** P fractionation in soils of Green Island chronosequence

Stage	Target P	Substrate	Extracted with	Procedure	Initial filtrate gives:	Digestion of filtrate with H_2_SO_4_, and H_2_O_2_ at 200 °C gives:
Pretreatment: Air dry, grind in agate mortar, sieve to 2 mm, weigh 0.5 g						
Non-sequential	Total P	Pre-treated fine earth	H_2_SO_4_ and H_2_O_2_	Digest sample in 97 % H_2_SO_4_, 30 %H_2_O_2_ & MgCl_2_ at 300^o^C	P_t_	
Sequential stage 1	Labile	Pre-treated fine earth	KCl	Swirl sample in 30 ml 0.1 M KCl for 30 min at room temperature	KCl Pi	
Sequential stage 2	Moderately labile inorganic a organic	Residue from Stage 1	NaHCO_3_	Swirl sample in 30 ml 0.5 M NaHCO_3_ for 16 h at room temperature	NaHCO_3_ P_i_	NaHCO_3_ P_t_
Sequential stage 3	Inorganic and organic on Fe and Al sesquioxides	Residue from Stage 2	NaOH	Swirl sample in 30 ml 0.1 M NaOH for 16 h at room temperature	NaOH P_i_	NaOH P_t_
Sequential stage 4	Calcareous and apatite	Residue from Stage 3	HCl	Digest sample in 15 ml 1 M HCL for 16 h room temperature	HCl Pi	
Sequential stage 5	Residual P Strongly adsorbed or occluded	Residue from Stage 4	H_2_SO_4_ and H_2_O_2_	Digest residue in 97 % H_2_SO_4_, 30 %H_2_O_2_ & MgCl_2_ at 300^o^C	Residual P	

Derivation of organic P in fractions: NaHCO_3_ P_o_ = NaHCO_3_ P_t_ - NaHCO_3_ P_i_; NaOH P_o_ = NaOH P_t_ - NaOH P_i_

Extracted P = Sum of extracts from Stages 1-4

Recovery % = (Extracted + Residual) / Total x 100

### Data analyses

The laboratory procedures for the fractionations of both sesquioxides and P require substantial time and effort. The potential for replication in studies like this is therefore limited, and *n* values, degrees of freedom, and the power of statistical analyses are low. As there were only single values for each sesquioxide fraction for each horizon, we used the means of the duplicate values for the P fractions for the correlations. For the statistical analyses we used the A horizon samples as the topsoils and the samples from the horizons showing the greatest pedogenic development, i.e. the most argic in the mid-altitude soils and most rubefied in the upper bench soils, as the subsoils.

The effect of bench height rank (which is taken as a surrogate for relative soil age) on general soil characteristics and pedogenic maturity, was examined by one-way analysis of variance for the sesquioxides, clay, pH and organic matter against bench height.

To examine for possible soil maturity trends in the sesquioxide and P variables, they were Spearman rank-based correlated against bench height/relative age. This was initially done for the whole data set, and then with the lower bench coral soils excluded.

To examine the associations between the sesquioxide and P variables, it was possible to estimate Pearson value-based correlations for all samples from the non-coral soils, and then separately for topsoils and for diagnostic subsoil horizons. All analyses were conducted using the Python sciPy, numPy, Pandas, and matplot.lib software packages language (Bezanson et al. 2017; https://www.scipy.org/citing.html).

## Results

### Sesquioxides

The initial one-way analyses of variance for Fe_d_, Fe_o_, Fe_d_-Fe_o_, Fe_d_-Fe_o_/Fe_d_, Al_d_, and Al_d_-Al_o_ against bench height rank are highly significant (p < 0.001), but only because of the substantial differences between the coral soils on the lower benches and the non-coral soils upslope. As our focus is on the effects of sesquioxides in the ferralisation chronosequence, further analyses are restricted to the non-coral soils on the benches at 50 m and above.

**Fig. 2:**
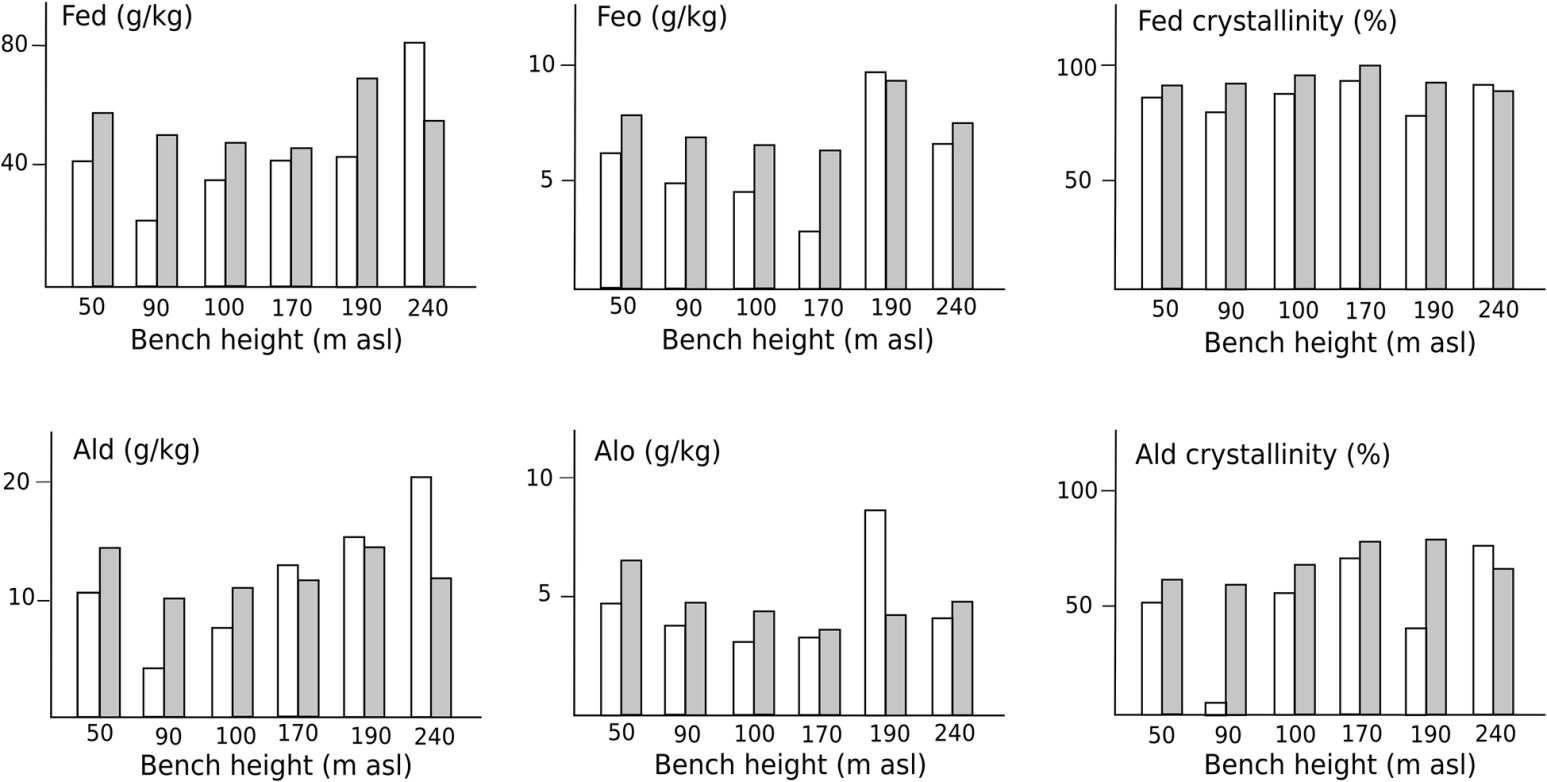
Sesquioxides (g kg^−1^) in non-coral top- and sub-soils in a ferralisation chronosequence on Green Island, Taiwan. Clear bars - topsoil; Shaded bars- diagnostic subsoil horizons

Total contents of iron (Fe_t_) were moderate to high (mostly > 50 g kg^−1^), with highest values in the oldest soil, Pedon 240 (Table 2), but the Spearman correlation with bench height rank is not significant. The contents of free pedogenic iron (Fe_d_) are moderate (> 20 g kg^−1^) throughout, but the intensity of iron weathering (Fe_d_/Fe_t_) is high, with Fe_d_ accounting for well over half of the total Fe_t_. Iron weathering intensity (Fe_d_/Fe_t_) increases slightly and erratically with bench height (Fig. 2) but the Spearman correlation is not significant. Within profiles, Fe weathering is most intense in the diagnostic subsoil argic (argillic) or ferralic (oxic) B horizons, with lower values in both the topsoils and weathering C horizons. Contents of amorphous Fe (Fe_o_) are low throughout, with a gradual decrease from 50 m up to 170 m, but are somewhat higher in the soils on the 190 and 240 m benches (Fig. 2). The low proportions of amorphous Fe_o_ imply that bulk of the free Fe (Fe_d_) is crystalline, shown by the high crystallinity ratios ((Fe_d_–Fe_o_)/Fe_d_) throughout, with the highest values in the oldest soils at 240 m, and the significant Spearman correlation (r = 0.504, p < 0.01) with bench height.

**Table 2:** Fe and Al extracts from soils of Green Island chronosequence

Bench height (m)	Horizon	pH	Organic C *(%)*	Fe_t_ Total Fe (g kg^−1^)	Fe_d_ Free Fe (g kg^−1^)	Fe_d_/Fe_t_ Fe freed (%)	Fe_o_ Non-crystalline free Fe (g kg^−1^)	Fe_d_ - Fe_o_ Crystalline free Fe (g kg^−1^)	(Fe_d_ –Fe_o_)/Fe_d_ Crystallinity of free Fe (%)	Al_t_ Total Al (g kg^−1^)	Al_d_ Free Al (g kg^−1^)	Al_d_/Al_t_ Al freed (%)	Al_o_ Non- crystalline free Al (g kg^−1^)	Al_d_ - Al_o_ Crystalline free Al (g kg^−1^)	(Al_d_ –Al_o_)/Al_d_ Crystallinity of free Al (%)
15	A	8.05	6.88	15.8	3.69	*23*	1.43	*2.26*	*61*	25.3	1.81	*7*	2.89	*− 1.08*	*–*
	C1	8.39	3.96	12.3	3.24	*26*	1.19	*2.05*	*63*	20.3	1.85	*9*	2.64	*− 0.79*	*–*
	C2	7.77	4.14	11.3	2.77	*25*	1.06	*1.71*	*62*	19.9	1.74	*9*	2.31	*− 0.57*	*–*
	C3	8.54	2.21	6.48	1.15	*18*	0.67	*0.48*	*42*	12.4	1.18	*9*	1.12	*0.06*	*5*
20	A	7.29	5.60	29.0	6.90	*34*	3.49	*3.41*	*49*	90	10.3	*11*	16.5	*− 6.2*	*–*
	Bw	6.79	0.79	23.0	5.80	*25*	3.15	*2.65*	*46*	71	6.72	*9*	2.49	*4.23*	*63*
	BC	6.84	0.19	nd	5.82	–	5.42	*0.40*	*7*	nd	2.69	*–*	2.99	*− 0.30*	*–*
	C	7.10	0.11	30.0	3.68	*12*	5.06	*− 1.38*	*–*	71	1.66	*2*	1.91	*− 0.25*	*–*
50	A	5.16	2.36	74.9	42.2	*56*	6.05	*36.2*	*86*	80.3	10.6	*13*	5.06	*5.5*	*52*
	Bt	5.00	1.61	73.8	46.8	*63*	6.97	*39.8*	*87*	88.2	10.8	*13*	6.00	*4.8*	*44*
	2 A	5.28	2.56	69.7	47.1	*68*	13.6	*33.5*	*71*	68.7	11.8	*17*	6.89	*5.0*	*42*
	2Bt	5.07	1.57	81.0	58.3	*72*	5.98	*52.3*	*90*	81.7	14.2	*17*	6.50	*7.7*	*54*
	2 C	4.61	0.23	67.0	36.0	*46*	8.27	*27.7*	*77*	108	9.53	*9*	8.43	*1.1*	*11*
90	A	5.04	3.28	64.5	22.0	*34*	4.72	*17.7*	*80*	53.4	4.39	*8*	4.11	*0.28*	*6*
	AB	5.28	2.50	63.5	49.5	*78*	5.72	*43.8*	*88*	63.4	10.3	*16*	4.96	*5.3*	*51*
	Bt1	5.18	1.73	77.8	51.0	*66*	4.68	*46.3*	*91*	84.0	9.79	*12*	4.68	*5.11*	*52*
	Bt2	5.28	1.30	69.6	46.4	*67*	5.18	*41.2*	*89*	63.8	8.83	*14*	4.58	*4.25*	*48*
	BC	5.16	0.30	78.4	33.9	*43*	4.92	*29.0*	*86*	84.5	6.90	*8*	4.87	*2.03*	*33*
100	A	6.44	2.73	59.3	35.2	*59*	4.34	*30.9*	*88*	84.3	7.65	*9*	3.31	*4.32*	*56*
	AB	7.42	2.01	53.7	38.1	*71*	4.75	*33.3*	*87*	74.0	7.91	*11*	3.36	*4.55*	*57*
	Bt1	7.44	0.77	62.0	46.2	*75*	2.68	*43.5*	*94*	81.7	9.23	*11*	3.33	*5.90*	*64*
	Bt2	7.15	0.88	59.6	48.2	*81*	2.54	*45.7*	*95*	64.2	10.9	*17*	4.38	*6.52*	*60*
	BC	6.90	0.31	44.8	23.7	*53*	1.38	*22.3*	*94*	71.5	6.47	*9*	3.54	*2.93*	*45*
170	O/A	4.02	2.84	68.1	41.7	*61*	2.62	*39.1*	*94*	nd	12.9	*–*	3.60	*9.30*	*72*
	Bt1	4.52	0.82	76.5	48.7	*64*	1.62	*47.1*	*97*	89.8	10.5	*12*	3.28	*7.22*	*69*
	Bt2	4.69	0.76	75.5	46.7	*62*	1.40	*45.3*	*97*	93.0	11.4	*12*	3.50	*7.90*	*69*
	Bt3	4.81	0.60	75.8	44.8	*59*	1.35	*43.4*	*97*	90.5	12.1	*13*	3.56	*8.54*	*58*
	Bt4	4.62	0.54	73.5	43.1	*59*	1.62	*41.6*	*97*	97.8	10.7	*11*	3.85	*6.85*	*64*
	C	4.62	0.25	58.6	23.1	*39*	1.85	*21.3*	*92*	101	5.66	*6*	3.42	*2.24*	*40*
190	A	6.01	7.80	nd	44.1	*–*	9.72	*34.4*	*78*	51.3	15.2	*30*	9.14	*6.06*	*40*
	2Bt1	4.75	1.32	68.6	61.3	*89*	9.51	*51.8*	*85*	78.0	13.1	*17*	4.97	*8.13*	*62*
	2Bt2	4.65	1.05	71.5	69.8	*97*	5.61	*64.2*	*92*	77.7	14.0	*18*	4.22	*9.78*	*70*
	2Bt3	4.56	0.80	78.3	60.3	*77*	5.19	*55.1*	*91*	96.3	18.7	*19*	4.37	*14.3*	*76*
240	A	4.78	7.40	93.9	82.2	*88*	6.53	*75.7*	*92*	60.8	20.2	*33*	4.40	*15.8*	*78*
	Bt1	4.62	1.25	94.8	54.9	*58*	4.93	*50.0*	*91*	97.2	11.5	*12*	4.79	*6.71*	*58*
	Bt2	4.71	0.86	77.2	57.4	*74*	3.20	*54.2*	*95*	71.6	12.1	*17*	4.65	*7.45*	*62*
	BC	4.62	0.53	98.9	55.9	*57*	2.71	*53.2*	*95*	89.5	11.3	*13*	4.44	*6.86*	*61*

t, XRF; d, dithionite-citrate-bicarbonate extract; o, oxalate extract, nd, no data

Total contents of aluminium (Al_t_) were similar to those for Fe_t_ (Table 2), and the Spearman correlation with bench height for the non-coral soils is also not significant. However, the weathering of Al follows a different trajectory from that of Fe, and the proportion of the liberated Al that remains free Al (Al_d_) is lower than for Fe, and the weathering intensity (Al_d_/Al_t_) therefore appears to be lower (Fig. 2). However, some of the Al released by weathering appears to co-precipitate with labile Si to form secondary aluminosilicates, particularly those with gibbsite interlayers, and some crystallizes out as gibbsite, particularly in topsoils (Jien et al. 2016). Aluminium in these forms does not register in the Al_d_ values. The contents of amorphous Al (Al_o_) are low, slightly lower than those of Fe_o,_ and show no discernible altitudinal trend. The crystallinities of the free Al_d_ ((Al_d_–Al_o_)/Al_d_) are high but substantially less than those for Fe_d_, but the Spearman correlation with bench height rank is highly significant (r = 0.641, p < 0.001). However, apart from crystallinity, the associations of the sesquioxide indicators of ferralisation with bench height/age are erratic, and the original designation of the ferralisation trend as ‘incipient’ was correct (Jien et al. 2016).

### P fractions

Total content of P (P_t_) is higher in the topsoils than in the diagnostic subsoil horizons (Table 3), probably due to biotic recycling (Shiau et al. 2018). Total P values rise again with depth in some saprolitic C horizon. Comparison of the diagnostic subsoil horizons in the non-coralline soils show substantial differences in P_t_ between profiles, but no altitudinal trend. The differences are attributed to minor but variable aeolian inputs of coral material from downslope and to lithological variations in the volcanic parent materials.

**Table 3:** P concentrations (mg kg^−1^) in the soils of the Green Island chronosequence

Bench height (m)	Horizon	Total Pt	KCl Pi	NaHCO_3_ Pi	NaHCO_3_ Po	NaOH Pi	NaOH Po	HCl Pi	Sum of extracts	Residual P	Overall Pi%	Overall Po%
15	A	564	3.20	12.6	79	9.16	225	120	449	75	32.3	67.7
	C1	477	2.11	9.15	84	6.28	163	125	390	285	36.6	63.4
	C2	471	1.80	9.80	81	5.30	145	127	370	235	38.9	61.1
	C3	213	0.85	4.44	82	3.71	142	123	356	65	37.1	62.9
20	A	783	1.43	24.4	133	138	489	129	915	424	32.0	68.0
	Bw	296	0.65	4.74	63	92	292	19.4	42	107	24.9	75.1
	BC	113	0.63	3.94	53	35	121	21.0	235	60.0	25.8	74.2
	C	322	0.61	4.05	67	28	111	284	555	75.0	67.9	32.1
50	A	536	0.68	11.7	86	195	271	1.97	567	430	36.9	63.1
	Bt1	475	0.35	5.65	97	145	257	2.31	507	381	30.2	69.8
	2 A	427	0.39	4.00	98	142	285	1.50	531	312	27.9	72.1
	2Bt	440	0.41	2.60	84	199	315	2.86	604	387	33.9	66.1
	C	1153	0.75	50.2	137	367	412	34.0	1001	934	45.2	54.8
90	A	209	0.70	6.56	98	48	150	2.01	306	183	18.9	81.1
	BA	221	0.36	3.62	68	39	136	2.13	248	164	18.1	81.9
	Bt1	178	0.46	2.75	64	29	120	3.22	220	159	16.2	83.8
	Bt2	187	0.36	2.60	64	28	102	3.90	201	155	17.4	82.6
	BC	156	0.49	3.35	61	28	59	3.39	156	142	22.8	77.2
100	A	458	0.65	6.36	79	64	170	3.51	324	309	23.2	76.8
	AB	441	0.68	5.18	53	47	120	6.70	233	342	25.6	74.4
	Bt1	269	0.33	2.77	44	62	113	2.09	225	187	30.1	69.9
	Bt2	228	0.34	2.69	64	50	97	3.91	217	216	26.4	73.6
	BC	202	0.35	3.01	58	31	59	5.54	157	169	25.4	74.6
170	A	320	0.42	19.5	36	83	188	5.25	332	292	32.5	67.5
	Bt2	264	0.16	1.72	39	77	154	6.02	278	298	30.5	69.5
	Bt2	287	0.27	1.92	39	85	151	6.59	284	314	33.0	67.0
	Bt3	291	0.22	2.27	39	90	148	4.48	283	316	34.0	66.0
	Bt4	314	0.02	2.40	38	88	177	5.30	310	387	30.9	69.1
	C	597	0.28	12.2	53	111	172	12.0	360	653	37.6	62.4
190	A	518	0.59	10.2	81	83	336	2.70	514	266	18.8	81.1
	Bt1	342	0.48	1.90	58	44	201	3.90	309	231	16.4	83.8
	Bt2	353	0.43	1.40	62	64	220	8.15	356	286	20.6	79.4
	Bt3	362	0.60	1.45	60	77	213	7.40	359	283	24.1	75.9
240	A	577	0.56	36.0	74	119	288	3.95	522	377	31.0	69.0
	Bt1	234	0.24	3.22	26	37	103	7.19	176	270	27.0	73.0
	Bt2	212	0.24	3.87	52	25	81	5.10	167	262	20.5	79.5
	BC	138	0.24	2.46	54	27	69	6.61	164	229	22.4	76.0

**Fig. 3:**
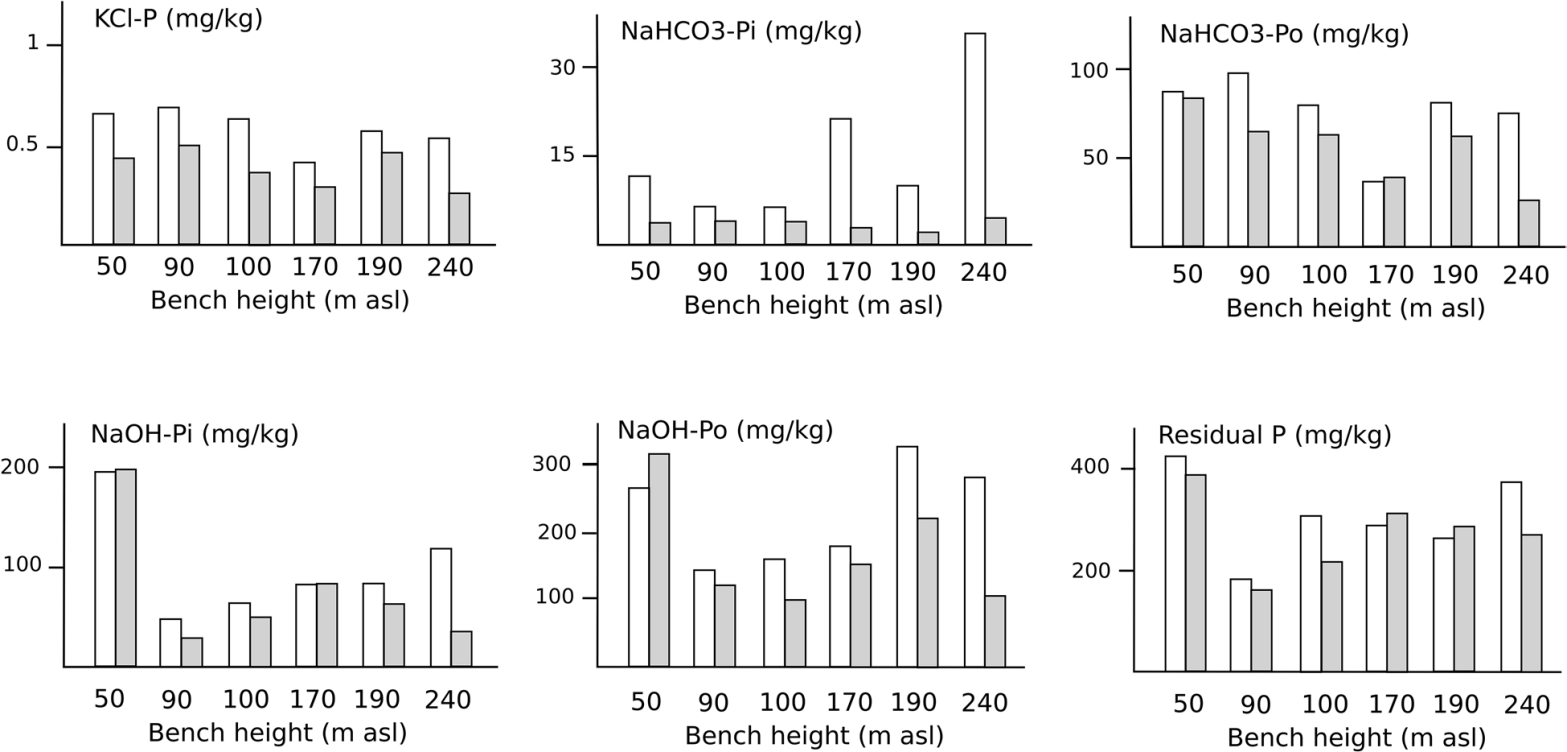
P fractions (mg kg^−1^) in non-coral top- and sub-soils in a ferralisation chronosequence on Green Island, Taiwan. Clear bars - topsoil; Shaded bars - diagnostic subsoil horizons

Although labile P, as extracted with KCl, is low throughout, values are consistently slightly higher in the topsoils, due to biotic recycling. The levels of P_i_ in the moderately labile NaHCO_3_ extracts are moderate, and are consistently lower than those from the NaOH extracts. The organic components of the NaOH extracts are consistently larger than the inorganic, by an order of magnitude or more. The preponderance of organic over inorganic components is also apparent in the NaHCO_3_ extracts, but the differences are less marked (Fig. 3). There are substantial quantities of residual P_r_. As in the other fractions, values are higher in the topsoils than in the diagnostic subsoil horizons, suggesting that even the least labile P is substantially organic. P_r_ values increase with bench height, indicating a moderate increase in P sequestration with age.

Although the coralline soils are not the main focus of this study, it is worth noting that their labile KCl-P values are as low as those in the non-coral soils, but their NaHCO_3_-P and NaOH-P values are considerably lower. They also have higher values in the topsoils than the subsoil, and a predominance of organic over inorganic forms. As expected, the P associated with calcareous soil components, as extracted with dilute HCl, is an order of magnitude greater than in the non-coral soils. Although lower than in the non-coral soils on the higher benches, residual P_r_ values are surprisingly substantial.

### P associations with sesquioxides

The Pearson correlations of the P fractions with the sesquioxides and other soil constituents for all of the non-coral samples (Table 4) show that the predominant organic forms of P in the NaHCO_3_ and NaOH extracts are significantly correlated with amorphous Fe_o_ and Al_o_. The importance of the amorphous sesquioxides is also apparent in the significant negative correlations for all of the topsoil and subsoil NaHCO_3_-P_o_ and NaOH-P_o_ extracts with the crystallinity of the free iron ((Fe_d_–Fe_o_)/Fe_d_) and, to a lesser extent, with that of free aluminium ((Al_d_ – Al_o_)/Al_d_). The inorganic P fractions are weak and correlated only with the crystallinities of Al_d_ and Fe_d_. The other sesquioxide variables, i.e. totals and total free contents, hardly register as significant correlates with any of the P fractions.

**Table 4:** Significant correlations between sesquioxides and P fraction in soils of Green Island chronosequence

	P fraction	KCl P	NaHCO_3_ Pi	NaHCO_3_ Po	NaOH Pi	NaOH Po	HCl P	Residual P
Soil attribute								
*All non-coral samples*								
Fe_t_	ns	ns	ns	ns	ns	ns	ns	ns
Fe_d_	ns	ns	ns	ns	ns	ns	ns	ns
Fe_t_/ Fe_d_	ns	ns	ns	ns	ns	ns	ns	ns
Fe_o_	ns	0.497**	ns	0.654***		0.64***	ns	ns
Fe_d_–Fe_o_	ns	ns	ns	ns	ns	ns	ns	ns
(Fe_d_–Fe_o_)/ Fe_d_	ns	– 0.639***	ns	– 0.773***	– 0.409*	– 0.558**	ns	ns
Al_t_	ns	ns	ns	ns	ns	ns	ns	ns
Al_d_	ns	ns	ns	ns	ns	0.449*	ns	ns
Al_t_/ Al_d_	ns	ns	ns	ns	ns	ns	ns	ns
Al_o_	ns	ns	0.39*	0.69***	0.565**	0.708***	ns	ns
Al_d_–Al_o_	ns	ns	ns	ns	ns	ns	ns	ns
(Al_d_–Al_o_)/ Al_d_	ns	-0.408*	ns	– 0.673***	ns	ns	ns	ns
Clay	ns	ns	ns	ns	ns	ns	-0.449	
pH	ns	ns	ns	ns	ns	ns	ns	ns
Organic C		0.423*	ns	ns	ns	ns	ns	ns
*Non-coral topsoils*								
Fe_t_	ns	ns	ns	ns	ns	ns	ns	ns
Fe_d_	ns	ns	0.929***	ns	ns	ns	ns	ns
Fe_t_/ Fe_d_	ns	ns	ns	ns	ns	ns	ns	ns
Fe_o_	ns	ns	ns	0.861*	ns	ns	ns	ns
Fe_d_–Fe_o_	ns	ns	0.955***	ns	ns	ns	ns	ns
(Fe_d_–Fe_o_)/ Fe_d_	ns	ns	ns	ns	ns	ns	ns	ns
Al_t_	ns	ns	ns	ns	ns	ns	ns	ns
Al_d_	ns	ns	0.856*	ns	ns	ns	ns	ns
Al_t_/ Al_d_	ns	ns	ns	ns	ns	ns	ns	ns
Al_o_	ns	ns	ns	ns	ns	ns	ns	ns
Al_d_–Al_o_	ns	ns	0.956***	ns	ns	ns	ns	ns
(Al_d_–Al_o_)/ Al_d_	ns	ns	ns	ns	ns	ns	ns	ns
Clay	ns	ns	ns	ns	ns	ns	ns	ns
pH	ns	ns	ns	ns	ns	ns	ns	ns
Organic C	ns	ns	ns	ns	ns	ns	ns	ns
*Non-coral subsoils*								
Fe_t_	ns	ns	ns	ns	ns	ns	ns	ns
Fe_d_	ns	ns	ns	ns	ns	ns	ns	ns
Fe_t_/ Fe_d_	ns	ns	ns	ns	ns	ns	ns	ns
Fe_o_	ns	ns	ns	0.82*	ns	ns	ns	ns
Fe_d_–Fe_o_	ns	ns	ns	ns	ns	ns	ns	ns
(Fe_d_–Fe_o_)/ Fe_d_	ns	– 0.862*		– 0.874*	ns	ns	ns	ns
Al_t_	ns	ns	ns	ns	ns	ns	ns	0.821*
Al_d_	ns	ns	ns	ns	ns	ns	ns	ns
Al_t_/ Al_d_	ns	ns	ns	ns	ns	ns	ns	ns
Al_o_	ns	ns	ns	0.892*		ns	ns	ns
Al_d_–Al_o_	ns	ns	ns	ns	ns	ns	ns	ns
(Al_d_–Al_o_)/ Al_d_	ns	ns	ns		0.952*	ns	ns	ns
Clay	ns	ns	– 0.936***	ns	ns	ns	ns	ns
pH	ns	ns	ns	ns	ns	ns	ns	ns
Organic C	ns	ns	ns	ns	ns	ns	ns	ns

ns: p> 0.05; *: p< 0.05; **: p< 0.01; ***: p< 0.001

Repeating the correlations for topsoils and the diagnostic subsoil horizons separately reduces the n values, degrees of freedom and significance of the correlations. However, separating the different parts of the profiles clarifies the P relationships, and r values are higher than for the mixed data (Table 4). The pattern of correlations in the topsoils differs from that in the mixed data. The inorganic component of the labile NaHCO_3_ extractable fraction is highly and significantly correlated with crystalline Fe_d_ and Al_d_. The correlation of the organic component of the same fraction with Fe_o_ has a high r value but only weak significance. There are no significant correlations with soil organic carbon.

The pattern of correlations in the subsoil is similar to that for the mixed data. This is to be expected as subsoil values make up most of the mixed data. The organic component of the NaHCO_3_ P fraction is correlated highly and significantly positively with Fe_o_ and Al_o_, and highly and significantly negatively with the crystallinity of the free iron ((Fe_d_–Fe_o_)/Fe_d_). However, there is no significant correlation with the crystallinity of the free Al_d_. As in the topsoils, there are no significant correlations with soil organic carbon, but the inorganic component of the NaHCO_3_ extract has a high and significant negative correlation with clay content in topsoils.

## Discussion

A study in Java showed that P retention is a serious problem for young volcanic soils, even where highly weathered and tending to ferralic (Sukarman et al. 2020). The chemical and mineralogical attributes of the sesquioxidic soils of Green Island indicate significant, if still incipient ferralisation. However, the soils straddle the middle regions of the Walker and Syers (1976) pedo-senility paradigm, with P being sequestered in both organic and mineral forms. Together with the slight rises of P_t_ in some saprolitic horizons that may be due to traces of unweathered apatite (Nezata et al. 2007), this suggests that the P-sequestration trajectory has progressed less that of the chemical and mineralogical indicators.

Nonetheless substantial proportions of the soil P are sequestered in the inorganic NaOH and residual fractions (Fig. 3; Table 4). These inorganic subfractions are correlated with the age-related increasing crystallinities of the free sesquioxides. It is assumed this P is strongly adsorbed onto, and occluded into the particles of the accreting and increasingly crystallised free sesquioxides (Walker and Syers 1976). This P is likely to be sequestered for long periods, possibly >10^5^ years, as its recycling is mainly affected by geological-scale weathering (Yang et al. 2013).

As quantities of highly available KCl-extractable P are very low, it appears that the limited quantities of labile P reside mainly in the substantial organic subfractions of the NaHCO_3_ and, to a limited extent, NaOH extracts. The preponderance of organic subfractions in these extracts has been noted in other tropical soils and coastal sand dune forest soils (Lin et al. 2018; Mirabello et al. 2012; Turner and Engelbrecht 2011). The importance of cryptic organic matter, the dark colours of which are masked by the intense rubefaction of the free iron sesquioxides, has been noted in deep ferralitic soils elsewhere in the tropics (Harper and Tibbett 2013). In our results, these P subfractions are not significantly correlated with soil organic carbon, and this P appears not to be simply adsorbed onto or incorporated into the chemical structures of free organic matter. In our study, these subfractions are correlated with amorphous Fe_o_ and Al_o_, even though these account for small proportions of the total free Fe and Al (Agbenin 1994; Singh and Gilkes 1991) (Fig. 2). These organic subfractions of P therefore appear to be sorbed onto and incorporated into complexes of organic matter and amorphous sesquioxides, especially Al_o_ (Wang et al. 2019). This P is more available than that associated with crystallising sesquioxides, but its recycling depends on the dynamics of the complexes, and the rate at which the organic components are decomposed (Barthes et al. 2008; Hernandez-Soriano 2012).

Reported turnover rates of Al-organo complexes vary considerably but the more stable seem to range in age from 500 to 5000 years (Hagerty et al. 2014; Lützow et al. 2008; Mahia et al. 2006; Xu et al. 2017). This makes the P within such complexes as unavailable over the life spans of individual trees, but some may participate in the centennial and millennial nutrient cycling of stable forests and affect succession trajectories.

## Conclusions

Soil macromorphology, chemistry and mineralogy indicate incipient ferralisation in the soils of the chronosequence, but the parallel trends in P sequestration are weak. Some of the P is sorbed into Al-organo complexes, which vary in their turnover rates. Some of the complexes persist for millennia and the recycling of their sorbed P is likely to be very slow. Although the increase in the quantities of sesquioxides with age are only slightly significant, their crystallinities increase with age. P occluded within the crystalline forms is likely to be completely immobilised, and to be recycled only within time scales of geological weathering. The current dynamics of P in the soils of the Green Island chronosequence appear to be mainly determined by complexes of amorphous Al and organic matter.

## Data Availability

Please contact author for data requests.

## Abbreviations

Al_d_: Free form aluminium oxide
Al_o_: Amorphous aluminium oxide
Al_t_: Total aluminium
Fe_d_: Free form iron oxide
Fe_o_: Amorphous iron oxide
Fe_t_: Total iron
P: Phosphorus
P_i_: Inorganic P
P_o_: Organic P
P_r_: Residual P
P_t_: Total P
XRF: X-ray fluorescence
